# NGF Enhances CGRP Release Evoked by Capsaicin from Rat Trigeminal Neurons: Differential Inhibition by SNAP-25-Cleaving Proteases

**DOI:** 10.3390/ijms23020892

**Published:** 2022-01-14

**Authors:** Mariia Belinskaia, Tomas Zurawski, Seshu Kumar Kaza, Caren Antoniazzi, J. Oliver Dolly, Gary W. Lawrence

**Affiliations:** International Centre for Neurotherapeutics, Dublin City University, Collins Avenue, D09 V209 Dublin, Ireland; mariia.belinskaia2@mail.dcu.ie (M.B.); tom.zurawski@dcu.ie (T.Z.); seshukumar.kaza@dcu.ie (S.K.K.); caren.antoniazzi@dcu.ie (C.A.); oliver.dolly@dcu.ie (J.O.D.)

**Keywords:** botulinum neurotoxins, trigeminal ganglion neurons, nociceptor sensitisation, nerve growth factor, capsaicin, calcitonin gene-related peptide, chimeric neurotoxin, TRPV1

## Abstract

Nerve growth factor (NGF) is known to intensify pain in various ways, so perturbing pertinent effects without negating its essential influences on neuronal functions could help the search for much-needed analgesics. Towards this goal, cultured neurons from neonatal rat trigeminal ganglia—a locus for craniofacial sensory nerves—were used to examine how NGF affects the Ca^2+^-dependent release of a pain mediator, calcitonin gene-related peptide (CGRP), that is triggered by activating a key signal transducer, transient receptor potential vanilloid 1 (TRPV1) with capsaicin (CAP). Measurements utilised neurons fed with or deprived of NGF for 2 days. Acute re-introduction of NGF induced Ca^2+^-dependent CGRP exocytosis that was inhibited by botulinum neurotoxin type A (BoNT/A) or a chimera of/E and/A (/EA), which truncated SNAP-25 (synaptosomal-associated protein with Mr = 25 k) at distinct sites. NGF additionally caused a Ca^2+^-independent enhancement of the neuropeptide release evoked by low concentrations (<100 nM) of CAP, but only marginally increased the peak response to ≥100 nM. Notably, BoNT/A inhibited CGRP exocytosis evoked by low but not high CAP concentrations, whereas/EA effectively reduced responses up to 1 µM CAP and inhibited to a greater extent its enhancement by NGF. In addition to establishing that sensitisation of sensory neurons to CAP by NGF is dependent on SNARE-mediated membrane fusion, insights were gleaned into the differential ability of two regions in the C-terminus of SNAP-25 (181–197 and 198–206) to support CAP-evoked Ca^2+^-dependent exocytosis at different intensities of stimulation.

## 1. Introduction

The neurotrophin, nerve growth factor (NGF), influences many functional aspects of neurons [1,2] with its intriguing roles in pain signalling being of particular interest [3,4]. Pain perception is usually initiated by specialised peripheral sensory neurons activated by noxious stimuli called nociceptors, mainly Aδ and C fibres. These are located in pairs of dorsal root ganglia (DRG), organised bi-laterally parallel to the spinal column, and in trigeminal ganglia (TG) plus several other smaller ganglia in the case of craniofacial nerves [5]. Peripheral projections from the nociceptors innervate virtually all organs and tissues of the body, which they monitor for the presence of potentially harmful chemical, thermal, and mechanical conditions. Via a range of different molecular sensors, such environmental factors excite nociceptor fibres to generate afferent action potentials that transmit signals via ascending pathways to the brain somatosensory cortex where pain is perceived [6]. One of the most intensively studied sensors expressed in nociceptors is the transient receptor potential vanilloid receptor 1 (TRPV1), a non-selective cation channel that is activated by noxious heat (>43 °C), protons and various chemicals including capsaicin (CAP), the component of chilli peppers responsible for causing heat sensation [7]. These activators open a channel pore in TRPV1 to permit an influx of Na^+^ and Ca^2+^. TRPV1 is the unique receptor for CAP, so the latter is a convenient tool for studying this channel and neurons that express it [8,9,10]. Tissue damage after injury or infection enhances the generation of pain signals by lowering the activation thresholds of nociceptors. This results in heightened sensitivity to painful stimuli (hyperalgesia) and the production of pain signals under normally innocuous conditions (allodynia). Inflammation-associated sensitisation involves the release of pro-algesic affectors from mast cells, macrophages, and sensory neurons at sites of injury or infection [11]. This normally serves to protect damaged tissues from further harm but, under certain pathological conditions, inflammation may persist even after the original trigger has been resolved. The resultant hypersensitivity of nociceptors can contribute to persistent chronic pain.

TRPV1-mediated nociception is implicated in heat hyperalgesia induced by inflammation [8,9], and has been identified as a target of signalling cascades activated by inflammatory mediators such as NGF, which modulates nociceptor sensitivity in addition to its classical developmental roles in the survival and differentiation of sensory and sympathetic neurons [4]. NGF levels are elevated in patients with chronic pain conditions such as osteoarthritis, lower back pain, interstitial cystitis, rheumatoid arthritis, spondylarthritis, and migraine [4,12]. Inflammatory and certain other cell types synthesise and release NGF under pathological conditions including, but not limited to, keratinocytes, mast cells and macrophages [3,4]. Notably, none of these cell types express detectable levels of NGF in their normal quiescent state in adults [4]. Nerve injury does not induce the upregulation of NGF expression in sensory neurons themselves [4]. Preclinical studies on rats showed that intra-plantar injection of NGF induces heat hyperalgesia within minutes attributable to sensitisation of peripheral nociceptors [13]. There are two receptors for NGF: tropomyosin receptor kinase A (TrkA) and p75 neurotrophin receptor [3,4], but TrkA-mediated signalling seems more critical in pain development because p75 knockout mice still develop acute mechanical and heat hypersensitivity after subcutaneous administration of NGF [14]. Prolonged activation of TrkA signalling enhances the expression of many genes encoding proteins and peptides linked to pain signalling, including TRPV1 and the calcitonin gene-related peptide (CGRP) [4]. Whilst NGF binding to TrkA promotes neuronal survival via activation of Ras and the downstream extracellular related kinases 1 and 2 (ERK 1/2), which involves retrograde transport of signalling endosomes, TrkA elicits a more immediate and local modulation of nociceptors via stimulation of phosphoinositide 3-kinase and phospholipase C [4]. Exposure of sensory neurons to NGF results in sensitisation to CAP within minutes, indicating an early potentiation of TRPV1 without altered gene expression [15,16,17,18]. In this regard, three mechanisms of NGF-induced acute TRPV1 potentiation have been described: modulation of TRPV1 channel-opening probability [19], prevention of agonist-induced desensitisation [20], and fast mobilisation of additional channels from intracellular stores to the neuronal membrane through regulated exocytosis [18,21,22]. All of those involve NGF-TrkA initiated signalling cascades that culminate in the increased phosphorylation of TRPV1, but at different sites. Phosphorylation at Y200 increases trafficking of TRPV1 to the cell membrane, whereas phosphate addition to S502 and S801 alters channel opening probability [18].

Most TRPV1-positive neurons express CGRP, and CAP is an established trigger for migraine attacks linked to its induction of CGRP release [12,23]. CGRP is a powerful vasodilator, instigator of neurogenic inflammation plus flare, and its elevated blood levels during migraine attacks [24] are suggestive of increased release in this prevalent painful condition. Paradoxically, prolonged activation of TRPV1 with agonists, CAP and civamide, can reduce headache pain by causing a durable denervation of fibres that express this channel, but their clinical use has been restricted due to the severity of on-target side effects of burning pain and lacrimation, whilst clinical trials with TRPV1 antagonists are ongoing [23]. An alternative migraine treatment that has gained traction is the targeted blockade of CGRP exocytosis. In fact, botulinum neurotoxin type A (BoNT/A), a potent and specific inhibitor of transmitter release [25,26] due to truncation and inactivation of SNAP-25 (synaptosomal-associated protein with M_r_ = 25k), received FDA approval (BOTOX^®^, a complex of BoNT/A and non-toxic proteins) for treating chronic migraine but not the episodic form [27]. This came after BOTOX^®^ injections were shown in certain patients to reduce the frequency and severity of headache episodes in the Phase III Research Evaluating Migraine Prophylaxis (PREEMPT) series of clinical trials [28,29]. Follow-up studies reported that patients who respond well to BOTOX^®^ display higher serum levels of CGRP prior to treatment than non-responders, and after treatment serum levels are significantly reduced in responders only [30]. However, even in responders, the frequency and severity of migraine attacks are only partially reduced. It is well established that the short truncation of SNAP-25 by BoNT/A (it removes only nine residues from its C-terminus) destabilises SNARE complexes but does not prevent their formation [31]; consequently, BoNT/A only retards Ca^2+^ dependent neurotransmitter release [32,33]. The protease of BoNT/E removes a larger C-terminal fragment (26 residues) and this is enough to prevent stable (i.e., SDS-resistant) SNARE complexes forming [31,32], but trigeminal ganglion neurons (TGNs) are insensitive to BoNT/E due to a paucity of glycosylated SV2A and SV2B, the essential protein component of its high-affinity neuronal receptor [32,34]. This impediment to the delivery of BoNT/E protease was circumvented by genetic engineering to create a recombinant chimera,/EA, having the SV2A,B and C binding (H_C_) portion of/A [35] fused to the translocation (H_N_) and protease light chain (LC) domains of/E [36]. In terms of the fraction of SNAP-25 cleaved, sensory neurons are almost as susceptible to/EA [32] as they are to BoNT/A [37], but with a smaller and less functional product created. Thus, a more complete blockade of CGRP exocytosis evoked by 1 µM CAP was achieved with BoNT/EA [32] compared to BoNT/A [37]. Hence, with the long-term goal of improving the therapeutic utility of such neurotoxins (reviewed by Rasetti-Escargueil and Popoff [38]), herein induction of Ca^2+^-dependent CGRP release from sensory neurons by NGF, and its Ca^2+^-independent enhancement of CAP-evoked neuropeptide exocytosis, were shown to be inhibited by/EA and to a lesser extent by/A. 

## 2. Results

### 2.1. Exposure to CAP Induces Concentration-Dependent Increases in Intracellular Ca^2+^ ([Ca^2+^]_i_) in Neonatal Rat TGNs In Vitro 

**Figure 1:**
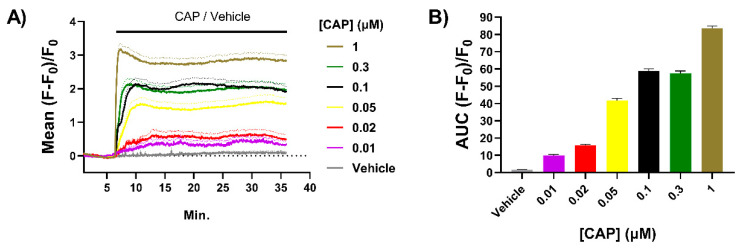
Capsaicin (CAP) induces dose-dependent increases of [Ca^2+^]_i_ in cultured trigeminal ganglion neurons (TGNs). Neurons were cultured for 4 days with nerve growth factor (NGF) before being loaded with Fluo-4 AM and recording fluorescence intensity by confocal microscopy. (**A**) Solid lines indicate the mean fluorescence (F) relative to initial fluorescence (F_0_), which was calculated [(F − F_0_)/F_0_] for cells exposed to different concentrations of CAP ([CAP]) and plotted against time. Broken lines indicate mean values +s.e.m. (standard error of the mean) n > 50 cells for each [CAP]. (**B**) Area under the curve (AUC) of mean fluorescence traces recorded for each [CAP] exposure. Column heights and error bars indicate mean AUC and s.e.m, respectively.

Cultured sensory neurons from neonatal rodents have been used extensively to study the potentiation by NGF of signalling by TRPV1 and related channels [15,21]. Compared to mature neurons from adult animals, these are relatively easy to isolate and cultivate at high density; also, ~85% of these neurons maintained in the presence of NGF express TRPV1 [39] and almost all of these co-express CGRP [37,40]. Binding of CAP causes the opening of a channel in TRPV1 that conducts Ca^2+^ (and other cations, mainly Na^+^) into neurons, and microscopic imaging of DRG neurons (DRGNs) or TGNs loaded with Ca^2+^-sensitive fluorescent dyes is a convenient method to study this nociceptive process [8,32]. Herein, TGNs were isolated from neonatal rats and cultured in the presence of NGF for 4 days before experiments. Just prior to confocal microscope imaging, cells were loaded with Fluo-4 AM as detailed in the Materials and Methods. After washing to remove unloaded dye, image recordings were begun with continuous superfusion of the recording buffer, and following a 6 min. period to record baseline signals, TGNs were exposed to CAP for 30 min. with recording continued throughout. A new sample of naïve cells was used for recording of responses to each CAP concentration ([CAP]) to avoid results being confounded by agonist-induced desensitisation. Increases in [Ca^2+^]_i_ were observed in 60% of the TGNs exposed to 0.01 μM CAP, the lowest concentration tested, rising to 85% of the neurons with 0.05 μM and remaining at about this level for higher [CAP], in good agreement with the fraction of TGNs found to express TRPV1 by immuno-histochemistry. The mean increase in fluorescence in responsive cells at each time point was calculated as a fraction of initial intensity (i.e., before exposure to CAP) and plotted + s.e.m. against time (Figure 1A). This highlights the escalation of [Ca^2+^]_i_ as [CAP] was raised. Note also that average fluorescence intensified more rapidly with each increment in [CAP] (Figure 1A). A plot of the area under the curve (AUC; Figure 1B) of mean fluorescence induced by each [CAP] over time illustrates a clear relationship between [CAP] and sustained increases in [Ca^2+^]_i_. These results accord with the [CAP] dose–response obtained by Ca^2+^-imaging in DRGNs [41]. By increasing [Ca^2+^]_i_ in TGNs due to activation of TRPV1 with CAP, exocytosis of the pain signalling neuropeptide CGRP [32] is triggered. Thus, it is of interest to elucidate the impact on CGRP release of factors known to modulate TRPV1 activity, such as NGF.

### 2.2. NGF Withdrawal from TGNs In Vitro Reduces Both Spontaneous and CAP-Evoked CGRP Release

Immature TGNs are highly dependent for survival on the presence of NGF in the culture medium and for eliciting the high expression of the signalling components [4]. Therefore, it was optimal to cultivate the neurons with this neurotrophin for some time before its withdrawal and later re-addition in order to examine the effects of re-introducing NGF on CGRP exocytosis, under resting conditions and upon activating TRPV1 with CAP. 

**Figure 2:**
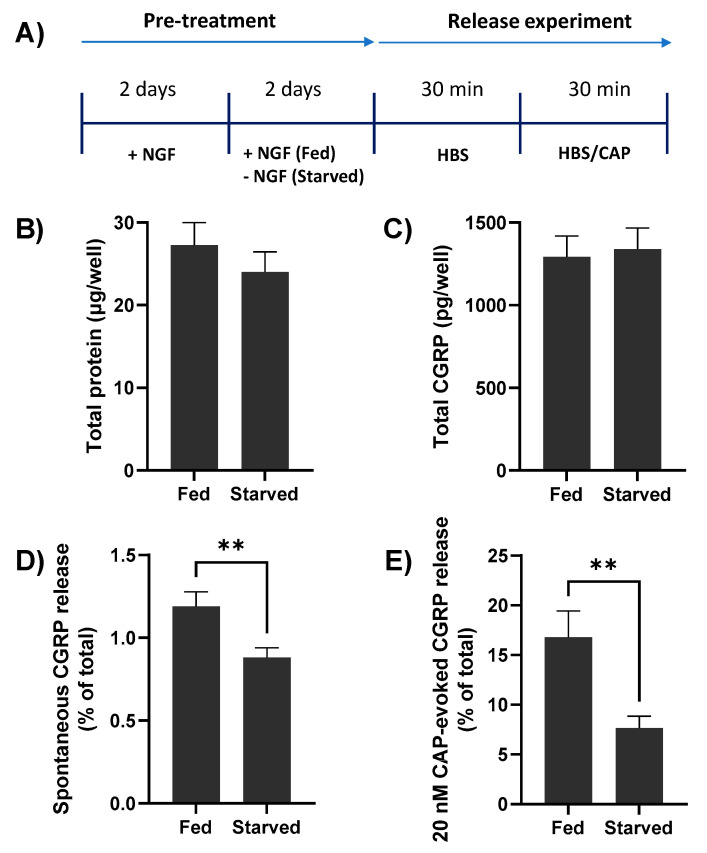
Deprival of NGF for 2 days does not significantly alter total protein or calcitonin gene-related peptide (CGRP) contents of rat cultured TGNs but reduces both spontaneous and CAP-evoked exocytosis of the neuropeptide. (**A**) Schematic illustrating the experimental protocol. Rat TGNs were cultivated initially in the presence of NGF (50 ng/mL) for 2 days before its withdrawal from half of the wells (starved) or retention for the other cohort over the 4 days (fed). The amounts of CGRP released were quantified during sequential 30 min. exposures, firstly to HEPES buffered saline (HBS) only ((**D**), spontaneous release), and then to 20 nM CAP in HBS (**E**). At the end of experiments, cells were solubilised with 1% (*v*/*v*) Triton X-100 in HBS, then total protein (**B**) and CGRP contents (**C**) were determined, as detailed in Materials and Methods. Data are presented as mean + s.e.m., n ≥ 12, N = 3. Asterisks summarise the results of unpaired *t*-tests with Welch’s correction, ** *p* < 0.01, shown only for significant differences.

TGNs were cultivated for 2 days in the presence of NGF (50 ng/mL) before transfer into a culture medium lacking this neurotrophin and were maintained for 2 further days (Figure 2A); anti-NGF antibodies were also added to neutralise any remaining traces. Note that some TGNs were continuously exposed to NGF for the entire 4 days (‘fed’) to serve as controls. Deprivation of NGF did not significantly change the total amount of protein present (27.3 ± 2.7 vs. 24.0 ± 2.4 µg/well [mean ± s.e.m.] in fed or starved cells, respectively; *p* = 0.38, Figure 2B), or their CGRP contents (1293 ± 125 [fed] vs. 1339 ± 127 [starved] pg/well; *p* = 0.8, Figure 2C), total TRPV1 expression (data not shown) and the proportion of cells expressing TRPV1 remained high (~74%) as determined by immuno-histochemistry. By contrast, spontaneous CGRP release was significantly lower for the starved neurons (0.88 ± 0.06% of total CGRP, c.f. 1.19 ± 0.09% in fed cells; *p* = 0.009, Figure 2D). Likewise, NGF removal decreased CGRP exocytosis evoked by 20 nM CAP (to 7.65 ± 1.2% of total CGRP, from 16.78 ± 2.6; *p* = 0.007, Figure 2E). In summary, starving TGNs of NGF for 2 days had no significant effect on the expression of total protein or CGRP but reduced the fraction of the latter released spontaneously or upon stimulation with a fixed low [CAP].

### 2.3. Depriving TGNs of NGF Reduces the Amount of CGRP Release Evoked by CAP 

**Figure 3:**
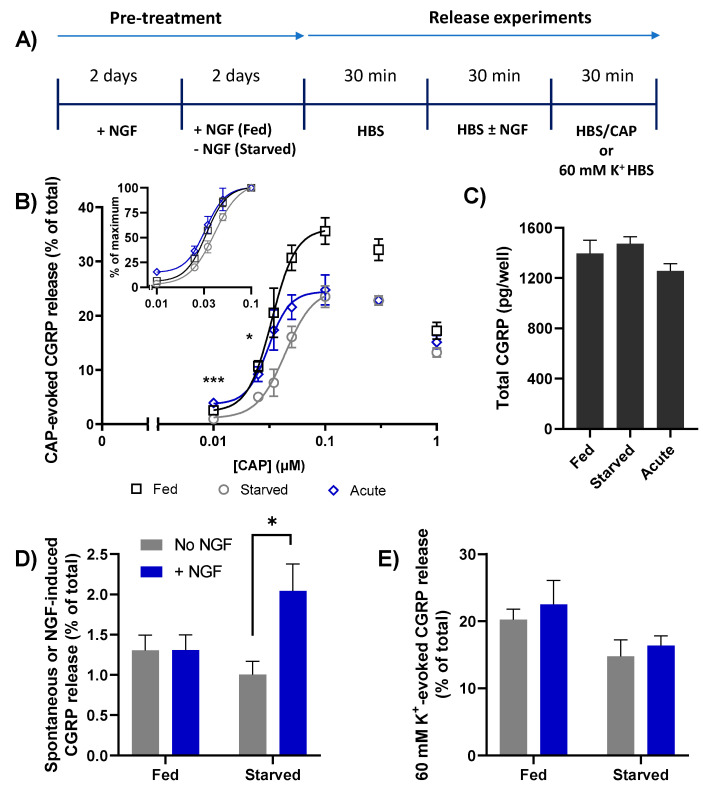
NGF withdrawal for 2 days from cultured TGNs reduces CGRP release stimulated by CAP; acute NGF induces exocytosis and enhances that stimulated with low [CAP]. (**A**) Timeline for pre-treatment and experimental manipulations of TGNs to determine the effect of NGF starvation and its brief re-introduction (100 ng/mL) on CGRP release under requisite conditions. (**B**) Dose-response relationship between [CAP] and CGRP release expressed as a % of total. Asterisks show significant differences between starved and NGF acutely treated cells (* *p* < 0.05, *** *p* < 0.001, n = 8, N ≥ 3, one-way ANOVA followed by Bonferroni’s post hoc test). Insert: The data obtained with 0.01–0.1 µM CAP replotted as a % of the requisite maximum release evoked by 0.1 µM. (**C**) Total CGRP contents. (**D**) Spontaneous or NGF-induced CGRP released from fed and starved cells (as indicated on abscissa) during the second of three 30 min. periods during which they were exposed to HBS only (grey bars) or HBS containing 100 ng/mL NGF (blue bars). Exposure of starved (but not fed) cells to NGF induced a significant increase in CGRP release (* *p* < 0.05, n = 9, N = 3, unpaired two-tailed Welch test). (**E**) In separate experiments, TGNs were incubated for 30 min. with/without NGF and then stimulated with 60 mM K^+^ HBS. Data is presented as mean + s.e.m.

To glean further insight into the consequences of NGF removal, the starved and fed neurons were stimulated with a range of increasing [CAP], using a slightly modified protocol (Figure 3A) to introduce a second 30 min incubation with HEPES buffered saline (HBS) prior to CAP stimulation. This extra step was to accommodate a further manipulation which is detailed later; otherwise, the procedure remained the same as in Figure 2A. In both fed and starved TGNs, stimulation with as little as 0.01 µM CAP produced a detectable amount of CGRP release above the baseline (i.e., spontaneous) level and increments in [CAP] produced increases that reached a peak at 0.1 µM (Figure 3B). Not only did further raising [CAP] fail to augment the amount of CGRP released from either fed or starved cells, in both cases a decline was observed with the higher [CAP]. Importantly, at all [CAP] tested the amount of CGRP release observed for NGF-starved cells was always less than the corresponding quantity seen with the fed neurons. The inclining phases of each dose response curve (0.01 to 0.1 µM [CAP]) were fit with four-parameter logistic functions to determine the maximum fraction of CGRP released from fed cells (37.3 ± 7.1% of total content); this was almost 1.5-times higher than the corresponding maximum evoked from starved cells (25.2 ± 2.1%) (Figure 3B). Notably, NGF withdrawal induced a small but just significant increase in EC_50_ values (32.6 ± 1.7 vs. 40.9 ± 1.8, *p* = 0.045). Thus, depriving neonatal rat TGNs of NGF for 48 h in vitro reduces their ability to exocytose CGRP in response to CAP and their apparent sensitivity to the TRPV1 agonist.

### 2.4. Brief Re-Exposure to NGF Induces CGRP Exocytosis from Starved TGNs and Augments the Amount Released in Response to Subsequent Stimulation with CAP

It has been established that brief exposure of previously-starved TGNs to NGF enhances CAP-evoked currents and increases [Ca^2+^]_i_ by augmenting TRPV1 activity [15,21]. So, the experimental protocol was modified to examine the consequence of acute NGF re-exposure of starved cells for CAP-evoked CGRP release. This involved a 30 min incubation of the cells with NGF (100 ng/mL) just prior to stimulating them with the various [CAP] described above (Figure 3A,B). Such an acute exposure to NGF did not significantly change the total CGRP content (Figure 3C), but it did elicit a two-fold increment in CGRP release compared to the spontaneous level in starved cells incubated with HBS alone (Figure 3D, *p* = 0.02). By contrast, in cells that had been continuously fed with NGF, the omission (or inclusion) of NGF during the 30 min recording period did not change the amount of spontaneous release (1.3% vs. 1.3% of total CGRP; Figure 3D). Presumably, the signalling events underpinning the aforementioned NGF-induced CGRP release in starved cells arose from the sudden return of a depreciated activity that was maintained constitutively in cells continuously fed with NGF. Immediately after the short application of NGF to the starved TGNs, they were exposed to a range of [CAP], from 10 nM–1 μM. Remarkably, acute treatment with NGF reversed the starvation-associated reduction in CGRP release evoked by low [CAP] (10 and 25 nM, *p* = 0.001 and *p* = 0.036 respectively, Figure 3B), was partially effective for intermediate [CAP] (35–50 nM), and caused a minor increase in CGRP release evoked from starved cells by higher [CAP] (0.1–1 µM). Fitting the inclining phase with a logistic function, as described before, confirmed that acute exposure of starved TGNs to NGF did not increase the maximum fraction of total CGRP that could be released upon stimulation with CAP (~25%) (Figure 3B). In contrast, the EC_50_ for [CAP] was lowered from 40.9 ± 1.8 nM (starved cells without acute NGF) to 31.6 ± 3.6 nM (starved cells after acute exposure to NGF), marking a restoration to the sensitivity displayed by TGNs fed continuously with NGF (EC_50_ = 32.6 ± 1.5 nM). Acute exposure to NGF selectively enhanced CGRP release triggered by activation of TRPV1 because the amount evoked by depolarisation with 60 mM K^+^ was not altered significantly in either fed or starved cells (Figure 3E). 

### 2.5. NGF Requires Extracellular Ca^2+^ for Inducing CGRP Release but Not Its Enhancement of 20 nM CAP-Evoked CGRP Exocytosis

**Figure 4:**
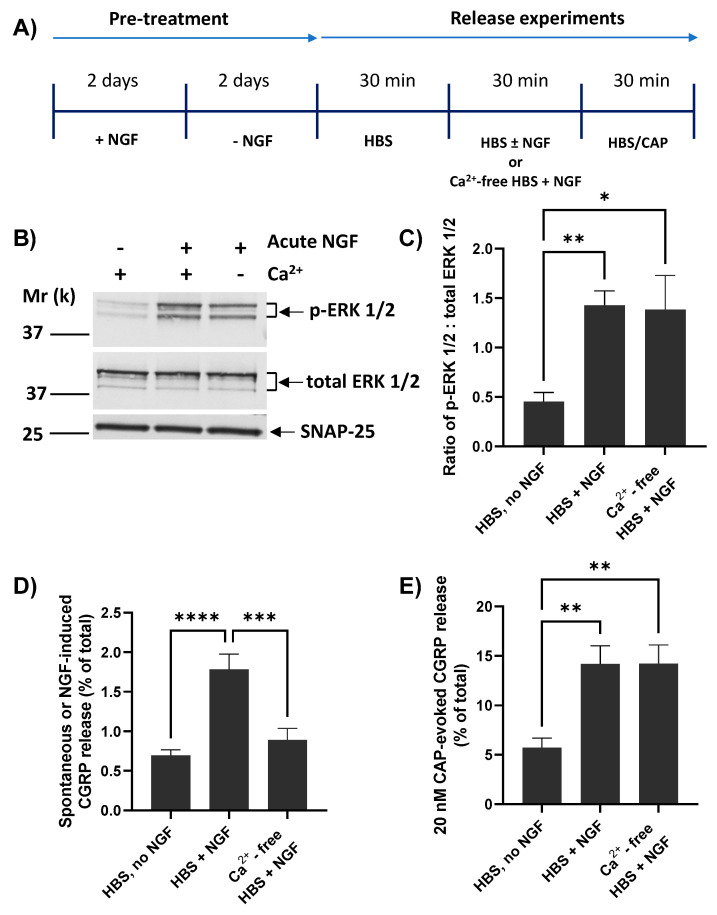
Extracellular Ca^2+^ is required for NGF to raise CGRP release but not for its enhancement of CAP-evoked Ca^2+^-dependent exocytosis. (**A**) NGF-starved TGNs were exposed in sequence for three 30 min. periods as follows. In the first period, all cells were exposed to HBS only. For period 2, the cells were split into three cohorts and incubated with Ca^2+^/HBS modified as follows: Cohort 1, HBS only; Cohort 2, Ca^2+^/HBS containing 100 ng/mL NGF; Cohort 3, HBS containing 100 ng/mL NGF but with Ca^2+^ replaced by 2 mM EGTA. In period 3, all cells were incubated with Ca^2+^/HBS containing 20 nM CAP. (**B**) In a separate set of experiments, TGNs were processed as far as period 2 before being lysed in 1x LDS buffer, and the ERK phosphorylation was determined by Western blotting as detailed in the Materials and Methods. (**C**) Histogram showing the ratio of signal intensity for p-ERK 1/2 to total ERK 1/2 (mean + s.e.m., n ≥ 3, N = 2), determined from the requisite immuno-reactive bands detailed in panel B. (**D**) Spontaneous or NGF-induced CGRP release during incubation period 2 and (**E**) 20 nM CAP-evoked CGRP release in period 3 calculated by subtracting the amount released during incubation 1, both expressed as a % of total CGRP content; n = 9, N = 3. For all histograms, one-way ANOVA was used followed by Bonferroni’s post hoc test, and significance indicated with asterisks; * *p* < 0.05, ** *p* < 0.01, *** *p* < 0.001, **** *p* < 0.0001. Each column and associated error bar represents mean + s.e.m., respectively.

Whilst CAP-evoked CGRP release from cultured TGNs is strictly dependent on the presence of extracellular Ca^2+^ [37], it seems that this is not the case for the enhancement by brief exposure to NGF of CAP-evoked TRPV1 currents and [Ca^2+^]_i_ accumulation [17,42]. Thus, the pertinent question of whether NGF-induced CGRP release requires Ca^2+^ was addressed using the protocol in Figure 4A. As described previously, TGNs were NGF starved before measuring spontaneous release (during the first of three 30 min. incubation periods) and then split into three cohorts. Two of these were exposed during a second 30 min. period to NGF, one of them in the presence of Ca^2+^ whilst the other was incubated without added Ca^2+^ and with 2 mM EGTA included to chelate any traces. The third batch was incubated without NGF but with Ca^2+^. After completing the latter stage, all three were then stimulated (a third 30 min. incubation) with 20 nM CAP in the presence of Ca^2+^ (which, as noted above, is essential for triggering CAP-evoked CGRP release). The absence of Ca^2+^ during acute exposure to NGF did not prevent its induction of ERK1/2 phosphorylation (Figure 4B). Indeed, a ~three-fold increase in the ratio of p-ERK1/2: total ERK 1/2 was observed relative to the proportion in continuously-starved cells (*p* = 0.02; Figure 4C), confirming effective activation of the NGF-TrkA signalling pathway. In fact, the latter ratio obtained in the absence of Ca^2+^ was indistinguishable from the proliferation of p-ERK 1/2 induced by NGF in the presence of Ca^2+^ (Figure 4C). By contrast, the absence of Ca^2+^ abolished the NGF-induced small increase in CGRP release relative to the spontaneous level (Figure 4D). Despite this, an approximately two and a half-fold enhancement by NGF of 20 nM CAP-evoked Ca^2+^-dependent CGRP release remained unaffected by the presence or absence of Ca^2+^ during the 30 min. that the cells were exposed to the neurotrophin (Figure 4E).

### 2.6. BoNT/A Blocks NGF-Induced Release of CGRP and Enhancement of That Stimulated by Low but Not High [CAP]

**Figure 5:**
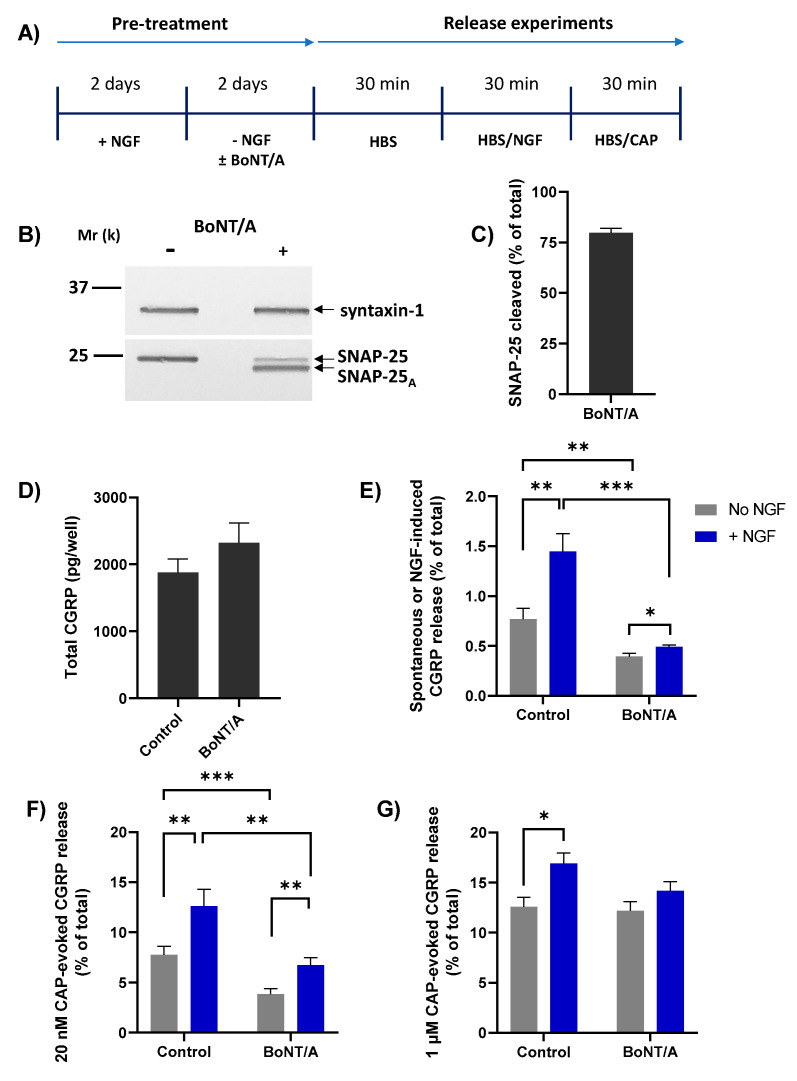
BoNT/A blocked NGF-induced CGRP release and -enhancement of 20 nM CAP-evoked CGRP release. (**A**) After 2 days in the presence of 50 ng/mL NGF, TGNs were starved of the neurotrophin as detailed before, without or with the inclusion of 100 nM BoNT/A during this latter step. The release experiment was performed as described in Figure 3A. (**B**) At the end of the protocol, one well each of BoNT/A-treated and non-treated cells were solubilised in 1× LDS and subjected to Western blotting. PVDF membranes were cut horizontally midway between the 25 k and 37 k molecular weight markers. The upper portion was exposed to antibodies reactive with syntaxin-1 (mouse monoclonal, 1:2000) and the lower piece probed with an antibody recognising both intact and BoNT/A-truncated SNAP-25 (mouse monoclonal, 1:3000). (**C**) The amount of cleaved SNAP-25 in BoNT/A-treated cells was calculated as a % (mean + s.e.m., N = 3) of the total SNAP-25 (intact + BoNT/A product). (**D**) Total CGRP (pg/well). (**E**–**G**) Histograms showing: (**E**) spontaneous CGRP release during the second 30 min. incubation into HBS without (grey bars) or induced by 100 ng/mL NGF (blue bars), (**F**) during the third period evoked by 20 nM CAP (minus the spontaneous release), and (**G**) during the third incubation with 1 µM CAP minus the spontaneous release. In all cases, CGRP release is expressed as a % of total CGRP (mean + s.e.m., N = 3, n = 9). Asterisks summarise the results of unpaired one-tailed Welch tests applied to the data plotted in panels E, F, and G, * *p* < 0.05, ** *p* < 0.01, *** *p* < 0.001.

The Ca^2+^ dependence of NGF-induced CGRP release suggests the involvement of regulated exocytosis. To determine whether this is SNAP-25 mediated, TGNs were treated with or without 100 nM of BoNT/A during the 2 days of NGF starvation (Figure 5A). This exposure of TGNs to BoNT/A for 48h led to cleavage of ~75% of the SNAP-25 present, as evidenced by the reduction in the amount of intact SNAP-25 and the appearance of a faster-migrating immuno-reactive band that was not observed in neurotoxin-free control cells (Figure 5B, C). Relative to control starved cells, BoNT/A-treated TGNs displayed a small increment in total CGRP content (Figure 5D) but this change was not significant (*p* = 0.22). On the other hand, basal neuropeptide release from the cells without NGF was nearly two-fold lower (Figure 5E, grey bars; 0.77 ± 0.11 vs. 0.40 ± 0.03% of total CGRP, mean ± s.e.m.; *p* = 0.005) compared to toxin-free control counterparts, revealing that spontaneous CGRP release largely entails SNAP-25-mediated exocytosis. Likewise, the NGF-induced elevation of CGRP release was significantly (*p* = 0.0003) lower in BoNT/A-intoxicated neurons than in toxin-free controls, confirming that this too arises from SNARE-dependent exocytosis (Figure 5E, blue bars). As before, acute treatment of starved TGNs with NGF induced a significant increment in the subsequent release evoked by 20 nM CAP (Figure 5F, c.f. control grey and NGF-treated blue bar, *p* = 0.006). BoNT/A reduced both CAP-evoked CGRP release from starved cells (Figure 5F, grey bars, *p* = 0.001) and its enhancement by acute treatment with NGF (Figure 5F, blue bars, *p* = 0.005). By contrast, BoNT/A did not reduce CGRP release stimulated by 1 µM CAP (Figure 5G, grey bars), and the enhanced level induced after pre-treatment with NGF was not lowered significantly (Figure 5G, blue bars).

### 2.7. Chimera/EA Inhibits CGRP Release Elicited by High [CAP]

**Figure 6:**
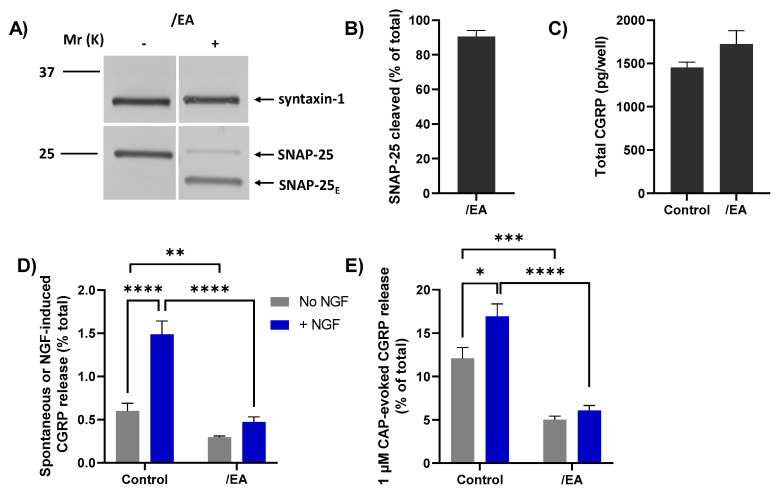
Chimera/EA effectively inhibits 1 µM CAP-evoked CGRP release from starved TGNs, and its enhancement by NGF. TGNs were starved and incubated with 100 nM/EA using a protocol identical to that described previously for BoNT/A (Figure 5A). (**A**) Western blotting with anti-SNAP-25 antibodies confirms the disappearance of intact SNAP-25 and the appearance of a much faster-migrating product in cells exposed to/EA (+) but not control (−). (**B**) Histogram displaying the % of SNAP-25 cleaved, which was calculated as in Figure 5C. (**C**) Total amounts (pg/well) of CGRP, determined as before, in control and/EA-treated cells. (**D**) Amounts of spontaneous CGRP release (% total) into HBS only, (grey bars), and that during incubation with 100 ng/mL NGF (blue bars), and (**E**) upon stimulation with 1 µM CAP (minus the spontaneous release) (mean + s.e.m. N = 3, n = 9). Unpaired one-tailed Welch test was applied to the data plotted in panels (**C**–**E**), * *p* < 0.05, ** *p* < 0.01, *** *p* < 0.001, **** *p* < 0.0001.

The/EA chimera was tested to ascertain whether it could cause a greater blockade than BoNT/A of CGRP release evoked by 1 µM CAP and its enhancement by acute NGF, using a protocol identical to the one described above for BoNT/A (Figure 5A). TGNs were starved of NGF and simultaneously incubated with 100 nM/EA before being briefly exposed to NGF (or control HBS) followed by stimulation with 1 µM CAP. Exposure to/EA resulted in the cleavage of more than 85% of the SNAP-25 present (Figure 6A, B), as reflected by the notably faster-migrating product (SNAP-25_E_) than that produced by BoNT/A (SNAP-25_A_; c.f. Figure 5B). As found for BoNT/A, total CGRP content was slightly, but not significantly, increased in EA-treated TGNs (Figure 6C) but both spontaneous CGRP exocytosis (Figure 6D, grey bars) and its elevation by NGF (Figure 6D, blue bars) were markedly reduced. Notably, pre-treatment with/EA reduced the 1 µM CAP-evoked CGRP release from NGF starved cells (Figure 6E, grey bars), unlike BoNT/A. Moreover, the enhancement of 1 µM CAP-evoked CGRP release observed in control cells after acute exposure to NGF (Figure 6E, control grey bar vs. control blue bar, *p* = 0.006) was absent in TGNs pre-treated with/EA. Consequently, this chimera clearly blocked responses to 1 µM CAP and its enhancement by NGF to a much greater extent than for BoNT/A (Figure 6E, c.f. Figure 5G). These results confirm the involvement of SNARE-dependent membrane trafficking in nociceptor sensitisation by NGF, a key mediator of inflammatory pain. 

## 3. Discussion

**Figure 7:**
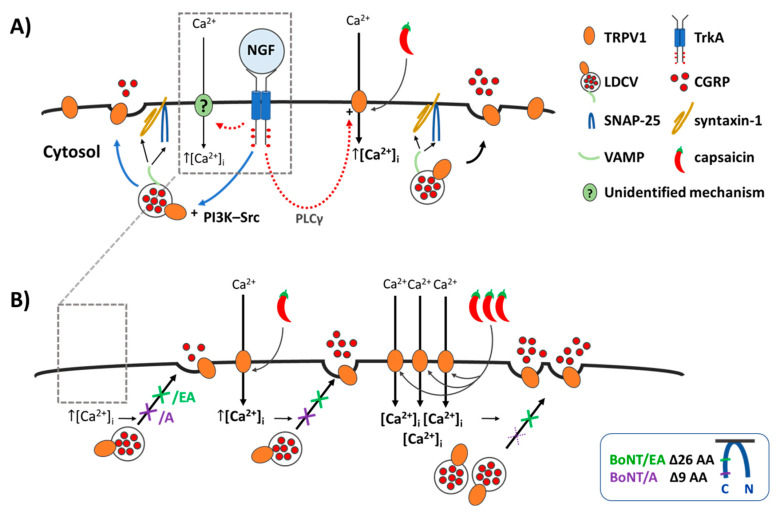
NGF induces a minor increase in Ca^2+^- and SNARE-dependent CGRP release, whereas it greatly enhances the CAP-evoked exocytosis which is blocked by BoNT/A and/EA at low [CAP] but only abolished by BoNT/EA at higher [CAP]. (**A**) Illustrates the effect of acute NGF on CGRP exocytosis from control neonatal rat TGNs starved of the neurotrophin for 2 days, and (**B**) in TGNs pre-treated with BoNT/A or /EA. (**A**) NGF binds to its receptor TrkA, activates the signalling cascades shown [18], and induces Ca^2+^ influx by an unidentified mechanism (**?**). Elevated intracellular Ca^2+^ ([Ca^2+^]_i_) triggers the fusion of large dense core vesicles (LDCVs) via SNARE-complexes (VAMP, syntaxin-1 and SNAP-25), thereby, causing exocytotic release of CGRP and surface delivery of vesicle constituents. This acute potentiation by NGF can involve the phosphatidylinositol 3-kinase—Src (PI3K-Src) pathway, which promotes trafficking of LDCVs, and insertion of their TRPV1 channels into the plasmalemma by Ca^2+^-regulated exocytosis (blue arrows) c.f. [18,21]. Additionally, the phospholipase C γ (PLCγ) cascade leads to sensitisation of TRPV1 already on the plasmalemma (red dashed arrows) [18]. The outcome of these composite influences of NGF on TRPV1 is that when the channel is activated by CAP **[Ca^2+^]_I_** is raised even more than normally [15,18,21] and this further enhances CGRP release (Figure 2). (**B**) The proteases of BoNT/A and/EA delete 9 (purple arrow) and 26 (green arrow) residues from SNAP-25 (Insert), respectively, preventing the fusion of LDCVs; this blocks the minimal CGRP exocytosis elicited by NGF (arrow with crosses, left) and its enhancement of the release evoked by 20 nM CAP (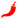) (arrow with crosses, centre). Stronger stimulation of TRPV1 with 1 µM CAP (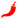

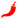

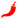
) induces a lot more Ca^2+^ influx (**[Ca^2+^]_i_**, (**[Ca^2+^]_i_**, (**[Ca^2+^]_i_**; Figure 1) which causes a moderate increase in CGRP release but overcomes the inhibition by BoNT/A (purple cross with broken lines, right) while/EA (green cross, right) remains effective in diminishing CGRP release. Acute sensitisation by NGF of TRPV1 selectively enhances neuropeptide exocytosis stimulated by low [CAP] (<100 nM) and only moderately affects responses to ≥100 nM CAP (Figure 3B). Despite being impotent against 1 µM CAP-evoked CGRP release in starved cells ((**B**),
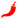

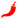

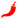
, and Figure 5G), BoNT/A partially inhibits the moderate NGF-enhancement of 1 µM CAP-evoked CGRP release (Figure 5G), implicating membrane trafficking in the sensitisation process (B, PI3K-Src stimulated pathway) in accordance with its inhibition of NGF-induced, Ca^2+^-dependent CGRP exocytosis (Figure 5E).

Amongst the factors released during chronic inflammation that contribute to persistent intransigent pain, NGF signalling has emerged as a prime candidate for therapeutic interventions [3,4]. NGF-sequestering monoclonal antibodies showed promise, but clinical trials had to be resumed with restricted dose protocols after serious adverse effects were detected in the original tests, apparently, due to interference with NGF’s roles in bone density maintenance and non-noxious sensation [3,4,43]. Thus, methods to mitigate NGF signalling that selectively target its pain-promoting pathways are needed. Exposure of sensory neurons to NGF causes an increase in their excitability and sensitivity to noxious molecules [4,15,16,17,19,44], with an involvement of membrane trafficking of nociceptive receptors to the neuron surface ([18,21]; Figure 7A). Moreover, the noxious chemical CAP activates its unique receptor TRPV1 [8,9], and induces increases in [Ca^2+^]_i_ [8,10] that trigger the exocytosis from receptive neurons of pro-inflammatory neuropeptides, substance P and CGRP ([37]; Figure 7A). In this regard, it is relevant to note that CGRP release from TGN fibres that densely innervate the meninges is strongly implicated in migraine, albeit not in all cases [45], and CAP is a recognised trigger of this painful condition (see Introduction). TRPV1-containing neurons are also clearly implicated in neurogenic inflammation, which can be induced by CAP injection. On the other hand, repeated application of the vanilloid prevents its induction of neurogenic inflammation due to targeted denervation of CAP-sensitive neurons [46].

The results of this in vitro study provide new insights into sensitisation of neonatal rat TGNs by NGF in relation to CAP-evoked CGRP exocytosis, as well as the different abilities of/A and/E proteases to inhibit the process (see later). Exposing NGF-fed TGNs to [CAP] from 20 to 100 nM yielded concentration-dependent increases in the amounts of both [Ca^2+^]_i_ (Figure 1A,B) and CGRP release (Figure 3B), which accords with reports of a four to five fold increment in [Ca^2+^]_i_ in FURA2-AM loaded cultures of neonatal rat DRGNs [41]. However, as reported by others using adult rat TGNs [47], further raising [CAP] to 300 nM and 1 μM caused a progressive reduction in the amount of CGRP exocytosed relative to the maximum level obtained with 100 nM (Figure 3B), unlike [Ca^2+^]_i_ which continues to accumulate in response to [CAP] up to 1 μM (Figure 1A,B) and as previously reported for DRGNs [41]. This suggests that the fall-off in CGRP release at high [CAP] (0.3 to 1 μM) is due to a curtailment of the stimulation of exocytosis at the higher [Ca^2+^]_i_ rather than any reduction in the responsiveness to high CAP concentrations arising from TRPV1 desensitisation, for example.

Neonatal TGNs are dependent on the presence of NGF for survival, cell attachment to the substratum and neuropil growth during the first 48 h in culture, so the effects of NGF deprivation were studied by removing the neurotrophin thereafter and maintaining the cultures in its absence for a further 48 h before experimentation, a commonly used protocol [15,21]. After 48 h of NGF deprivation, the amount of CGRP release evoked was depressed at all [CAP] relative to the corresponding levels from fed cells. Notably, the extent of reduction was greatest for 0.1 μM, which (like in fed cultures) also gave the peak amount of CGRP release in the starved TGNs. Importantly, starvation did not impair the expression of CGRP, so the latter must reflect a depressed ability of CAP to stimulate neuropeptide exocytosis from the cells deprived of NGF. Consistent with reports that brief exposure of starved DRGNs to NGF just prior to experimental recordings enhances CAP-induced currents and augments increases in [Ca^2+^]_i_ [15], it is shown here that acute re-introduction of NGF elevates CAP-evoked CGRP release from starved TGNs, but principally for [CAP] below 100 nM. As NGF is known to enhance currents elicited by 30–300 nM CAP and escalate the increases in [Ca^2+^]_i_ induced by 100 or 500 nM CAP [15,18,21], it is apparent that 100 nM CAP induces adequate [Ca^2+^]_i_ for optimum triggering of CGRP release from TGNs in vitro, and that this relationship is unaltered by NGF-starvation. Further increases in [Ca^2+^]_i_, which can be achieved using higher [CAP] [41] or acute exposure to NGF of starved neurons [15,18,21], are unable to increase CGRP release above the level evoked by 100 nM CAP because the latter already reached the optimum level of [Ca^2+^]_i_ for exocytosis. On the other hand, at [CAP] less than 100 nM the lower amounts of Ca^2+^ entry triggered by the vanilloid are sub-optimal for stimulating CGRP release (Figure 3B), so NGF-induced increases in CAP-evoked currents [42] and, consequently, [Ca^2+^]_i_ do boost CGRP release (Figure 3B; [48]). The outcome of NGF re-introduction to starved cells, therefore, is an apparent elevation in sensitivity to CAP (Figure 3B) because the boost to CGRP release, the consequence of augmented Ca^2+^ entry, is analogous to the growth in exocytosis of the neuropeptide observed when [CAP] was increased. Thus, the apparent change of sensitivity to CAP can be explained without there being an actual alteration in affinity of the vanilloid for its receptor, TRPV1; hence, plotting normalised CGRP release (as a% of the maximum level elicited by 100 nM) indicated that there are minimal differences in actual CAP sensitivity between NGF-fed, -starved and -starved then acutely fed TGNs (Figure 3B insert).

That re-introduction of NGF did not reverse the suppression by starvation of maximum attainable CGRP release (i.e., elicited by 100 nM CAP) despite no reduction in CGRP expression is suggestive that continuous presence of NGF in fed cells must increase the fraction of total CGRP pool available for exocytosis. As the starvation protocol also reduced the amount of CGRP released in response to depolarisation with 60 mM K^+^, albeit not significantly (Figure 3E), targets of NGF signalling other than TRPV1, such as voltage-gated channels or mediators of neuropeptide exocytosis (reviewed by [3,4]), likely contribute to the maintenance of high levels of CGRP release upon long-term (i.e., 2 days) exposure to NGF. Brief re-introduction of NGF seems to enhance recruitment by lower [CAP] (<100 nM) of the smaller fraction of CGRP that remains available after starvation, but cannot recover the increment that appears to be lost during starvation. By contrast, acute (30 min.) exposure to NGF had no effect on 60 mM K^+^-depolarisation evoked CGRP release in either fed or starved cells (Figure 3E). Together, these results highlight a specific fast stimulatory action of NGF on CAP-evoked CGRP release, thereby, implicating TRPV1 as a rapidly modified target of NGF signalling in accordance with current thought [3,4]. There are various ways by which NGF can modulate TRPV1 activity to boost CAP-stimulated Ca^2+^ entry (Figure 7A), including altering channel gating and reducing desensitisation [20]. However, the main contribution seems to be trafficking of TRPV1 from intracellular organelles (Figure 7A) to increase their density on the cell surface [18] and/or replace desensitised forms [49]. NGF-induced trafficking of TRPV1 via SNARE-mediated membrane fusion in DRGNs has been evidenced using a peptide inhibitor patterned after SNAP-25 [21], whilst immunocytochemistry revealed TRPV1 (and TRPA1) co-expression on large-dense core vesicles (LDCVs) that store CGRP [40,50] and also contain VAMP1 (Figure 7A) as well as synaptotagmin 1 [40]. Moreover, exposure of TGNs for 24 h to tumor necrosis factor alpha induced co-traffic of TRPV1 and TRPA1 to their plasma membrane, and this process was blocked by BoNT/A [40]. Also, the demonstrated requirement herein for the presence of extracellular Ca^2+^ for acute exposure to NGF to stimulate CGRP release from starved TGNs (Figure 4D), and its blockade by BoNT/A (Figure 5E) or/EA (Figure 6D), evinced an involvement of Ca^2+^- and SNAP-25-dependent LDCV exocytosis (Figure 7B). This accords with reports that acute exposure to NGF induces [Ca^2+^]_i_ signals in adult mouse DRGNs [51], and that in PC 12 cells this neurotrophin elicits a small [Ca^2+^]_i_ rise [52,53] and evokes catecholamine release that is dependent on extracellular Ca^2+^ (reviewed by [54]). Thus, in principle, the sensitisation of sensory neurons to CAP caused by NGF may involve the transfer of TRPV1 on LDCVs to the plasmalemma. However, it must also be considered that even in the absence of extracellular Ca^2+^ acute exposure of DRGs to NGF activates TrkA signalling (exemplified here by increased ERK1/2 phosphorylation; Figure 4B,C) and increases CAP-evoked Na^+^ currents indicative of enhanced TRPV1 activity [42]. Herein, this was corroborated by the finding that re-introduction to starved cells of NGF in the absence of Ca^2+^ still enhanced subsequent CGRP release triggered by 20 nM CAP. It was necessary to include extracellular Ca^2+^ alongside CAP to enable the stimulation of CGRP release but, nevertheless, these novel results clarify that even in the absence of CGRP exocytosis, starved TGNs are sensitised by NGF and subsequent responses to CAP are exaggerated. Such sensitisation might involve phosphorylation of TRPV1 or association of the channel with one or more other signalling molecules [4,18,44]. However, even a low [CAP] such as 20 nM evokes far more CGRP release than acute NGF (Figure 4E c.f. D), so it must cause much more transfer of TRPV1 on LDCVs to the plasma membrane ([50]; Figure 7B). Perhaps in the absence of extracellular Ca^2+^, NGF modifies TRPV1 to improve retention of the channel at the cell surface, rather than directly stimulating its transfer, or promotes docking of LDCV containing TRPV1 in advance of Ca^2+^-triggered fusion. The results presented here do not exclude that a component of NGF sensitisation to CAP occurs without trafficking of supplementary TRPV1 to the plasma membrane [18] but the extensive inhibition of the sensitisation by SNAP-25-cleaving BoNTs, particularly by/EA, accords with evidence that delivery of TRPV1 to the cell surface is the major factor [18].

The findings reported here clarify a possible basis for the prevention of nociceptor sensitisation by BoNT/A that may be relevant to its limited analgesic action in migraineurs with elevated CGRP (see Introduction); this notion arises because of its blockade of CAP-evoked CGRP release and of the enhancement by NGF, but only under conditions of relatively mild nociceptor activation (Figure 7B). The declining ability of BoNT/A to reduce neuropeptide exocytosis elicited by high [CAP] despite extensive proteolysis of SNAP-25 (75% of the cells’ complement) is likely related to the larger increase in [Ca^2+^]_i_ induced, relative to the respective signal brought about by 20 nM CAP (Figure 1A,B and [41]), and the prolonged persistence of raised [Ca^2+^]_i_ during 30 min. exposure to high concentrations of the vanilloid (Figure 1A and [32]). A similarly extensive proteolysis of SNAP-25 was observed for BoNT/EA, but with production of the shorter, more functionally disabled, product typical for/E (SNAP-25_E_), (Figure 6A,B). Strikingly, the latter was accompanied by large reductions in CGRP release evoked by either high [CAP] (Figure 6E) and the augmentation by NGF of exocytosis was prevented (Figure 6E), highlighting the superiority of BoNT/EA relative to/A for attenuation of CGRP release under strong stimulation conditions represented by 1 μM CAP (Figure 7B). As other nocisensitive channels implicated in migraine, TRPA1 [55] and P_2_X_3_ [56] also reportedly transfer to the surface of sensitised nociceptors, BoNTs could potentially provide more broadly effective analgesia than selective antagonism of any single channel. It is also suggested that modified BoNTs with/E protease activity might be a more effective option for sufferers exhibiting exceptionally excessive neuropeptide secretion, particularly if caused by over-excitable sensory neurons with high [Ca^2+^]_i_ loads [32]. Moreover, the recombinant engineering utilised here facilitates further potentially beneficial improvements such as to prolong the/E protease lifetime [57] and improve selectivity for sensory relative to other peripheral neurons [58].

## 4. Materials and Methods

### 4.1. Materials

NGF 2.5S and antibodies to NGF (AN-240) and TRPV1 (ACC-030; used to determine TRPV1 expression by Western blotting) were supplied by Alomone Labs (Jerusalem, Israel). Culture 48-well plates were purchased from Thermo Fisher (Cheshire, UK). Collagenase, Dispase^®^ and B-27^TM^ Supplement were bought from Bio-Sciences (Dublin, Ireland). Monoclonal antibodies specific for SNAP-25 (SMI-81) and syntaxin-1 (clone HPC-1; S0664), were obtained from Covance (now Labcorp Drug Development, Princeton, NJ, USA) and Merck (Arklow, Ireland) respectively. Antibodies raised in rabbits and specific for ERK1/2 (9102) and phosphorylated ERK1/2 (9101) were purchased from Cell Signalling Technology (Leiden, The Netherlands). Guinea pig anti TRPV1 (AB5566) was obtained from Millipore (Tullagreen, Ireland). Goat secondary antibodies reactive with mouse (A3688) or rabbit (A9919) IgG and conjugated with alkaline phosphatase (AP) were supplied by Merck. Western blotting reagents, polyvinylidene fluoride membrane (PVDF) and Bio-Rad protein standards were bought from Accu-Science (Kildare, Ireland). Lithium dodecyl sulphate (LDS) sample buffer and 12% BOLT™ Bis-Tris polyacrylamide gels were from Bio-Sciences. A wheat germ agglutinin Alexa Fluor 633 conjugate, Fluo-4AM and ProLong™ Glass Antifade Mountant were supplied by Thermo Fisher Scientific (Dublin, Ireland). Enzyme-linked immunosorbent assay (ELISA) kits for the detection and quantification of CGRP were purchased from Bertin Technologies (Montignyle Le Bretonneux, France). All other reagents used were obtained from Merck.

### 4.2. Isolation and Culturing of Rat TGNs

The animal husbandry and scientific procedures were approved on 1 May 2018 by the Research Ethics Committee of Dublin City University (DCUREC/2018/091). TGNs were dissected from 3 to 6 day-old Sprague Dawley rat neonates as described [37] and kept in ice-cold Ca^2+/^Mg^2+^-free Hanks’s balanced salt solution. After digestion with a 1:1 (*v*/*v*) mixture containing 1275 units (U) collagenase I and 17.6 U Dispase^®^ for 30 min. at 37 °C, 12.5 U of Benzonase^®^ nuclease was added to the mixture to reduce viscosity and clumping of the tissue; cells were gently agitated by trituration with a 2.5 mL Pasteur pipette during and after a further 15 min. incubation at 37 °C. To separate the neurons from unwanted non-neuronal cells, myelin and nerve debris, the suspension of dissociated cells was centrifuged through a discontinuous Percoll^®^ gradient as described in [59]. The resultant cell pellets were re-suspended in Dulbecco’s Modified Eagle Medium (DMEM) containing 10% (*v*/*v*) foetal bovine serum, 1% (*v*/*v*) penicillin-streptomycin, B-27^TM^ Supplement and 50 ng/mL 2.5 S NGF. TGNs were seeded at a density of ~20,000–30,000 neurons per well in 48-well plates that had been pre-coated with poly-L-lysine (0.1 mg/mL) and laminin (10 µg/mL). To suppress the growth of dividing (i.e., non-neuronal) cells, 10 μM of cytosine arabinoside (Ara-C) was added on day 1 and included for 5 consecutive days; the medium was changed every day, unless otherwise specified.

### 4.3. NGF Withdrawal from TGNs and Treatment with BoNTs

After 2 days in vitro (DIV), cells were washed thrice with 0.5 mL per well of standard DMEM-based medium but lacking NGF and containing 500 ng/mL anti-NGF antibodies, and 10 μM Ara-C. For the next 2 days, TGNs were maintained in this starvation medium or with the inclusion of 100 nM BoNT/A or /EA [36]. On DIV4, spontaneous, NGF-induced and CAP-stimulated CGRP release were quantified from these cells under the different conditions specified.

### 4.4. Intracellular Ca^2+^ Imaging

TGNs were prepared and cultured as described in 4.2 but were plated on 13 mm glass coverslips coated with poly-L-lysine and laminin. After 4 DIV, cells were washed with HEPES buffered saline (HBS, mM: 22.5 HEPES, 135 NaCl, 3.5 KCl, 1 MgCl_2_, 2.5 CaCl_2_, 3.3 glucose) supplemented with 10 µg/mL bovine serum albumin (HBS-LB) and loaded with 3 µM fluo-4-acetoxymethyl ester (Fluo-4 AM) in the presence of 0.02% pluronic F-127 acid for 20–30 min. at 37 °C. Cells were then placed in a superfusion chamber (RC-25; Warner Instruments, Holliston, MA, USA) mounted on the stage of a Zeiss LSM710 confocal microscope and left for 10 min. with 2 mL/min continuous perfusion with HBS-LB to equilibrate. Confocal imaging was performed at ambient temperature (22 °C) using a 488 nm argon laser and 20× magnification objective (EC Plan-NEOFLUAR/0.5 NA) at 0.33 Hz frame rate. Baseline fluorescence was recorded for 6 min before switching to HBS-LB containing CAP, which was added from 10,000× stock solutions prepared in ethanol on the day of use, and continued recordings in the presence of CAP for 30 min. Control recordings were performed with a vehicle (HBS-LB containing 0.01% (*v*/*v*) ethanol). At the end of each experiment, after washing out the CAP or vehicle, TGNs were stimulated with 100 mM KCl in HBS-LB (isotonically balanced by reducing the [NaCl]) to determine the total number of viable Fluo-4 AM loaded TGNs in the image field.

The intensities of recorded fluorescence signals (F) were analysed offline. Regions of interest (ROIs) were applied to individual TGN somata, and F was measured for each ROI in every frame of the video recordings using the average pixel intensity tool in Image J (version 1.53e, National Institutes of Health, USA). Values were exported to Microsoft Excel^®^ (Office 365, Microsoft Corporation, St Redmond, WA, USA) for further analysis. Measurements for time-points recorded during the first 6 min were averaged to determine initial fluorescence intensity (F_0_) and the standard deviation (s.d.) in this baseline signal over this period. Changes in fluorescence intensity (F) relative to initial values (F_0_) were calculated for every time point using the formula (F − F_0_)/F_0_. ROIs that exhibited an increase of fluorescence such that (F − F_0_)/F_0_ was greater than F_0_ plus 10× s.d. were considered to be responders. Mean (F − F_0_)/F_0_ values and the s.e.m. were determined for each time point from all responders and plotted against elapsed time using GraphPad Prism 9 (GraphPad Software, San Diego, CA, USA). The AUC of traces generated for each [CAP] or vehicle were determined using the tool available in the latter program.

### 4.5. Incubation of TGNs to Monitor CGRP Release

All release of CGRP was determined after 30 min. incubation at 37 °C using 0.25 mL per well of HBS containing 0.1% bovine serum albumin (HBS-BSA; except for the measurement of spontaneous release, stimulants were added). Note that for some experiments, CaCl_2_ was omitted from the HBS and replaced with 2 mM EGTA, as detailed in the relevant Figure legends. For stimulation with CAP, working dilutions (in ethanol) were prepared on the day of use from a 100 mM stock stored in ethanol at −20 °C and diluted to the required concentration in HBS-BSA; as a control for the solvent, 0.1% ethanol was included in the HBS-BSA. CGRP release was also stimulated with 60 mM KCl in HBS-BSA (isotonically balanced with reduced [NaCl]). At the end of each experiment, all remaining fluids were aspirated and the cells dissolved in 1% Triton X100/HBS, kept on ice for 10–15 min. and triturated through a 1 mL pipette tip to maximise the disruption of the plasmalemma and LDCV membranes. All aliquots of HBS-BSA were removed after the various incubations, and the solutions of detergent-lysed cells, were centrifuged (20,000× *g*) for 1 min. at 4 °C to remove insoluble matter. The supernatants were stored at −20 °C until the day of assay. 

### 4.6. Quantification of CGRP by ELISA

To quantify CGRP, 0.1 mL of each sample was added to 96-well plates coated with a monoclonal antibody against CGRP. ELISA was performed following the manufacturer’s instructions. A series of diluted CGRP standards was included every time an assay was performed, and a standard curve (linear fit) generated in Graph Pad 9. CGRP concentrations in samples were determined by reference to this standard curve. Any samples giving values out of the standard range were re-analysed after appropriate dilutions. Calculations were performed using MS Excel. Spontaneous release values were subtracted from those obtained for the same well upon stimulation by CAP or K^+^ depolarisation to yield the evoked component. To facilitate comparisons between experiments, released CGRP was normalised as a % of total CGRP (i.e., the sum of released CGRP (from several experimental steps, where applicable) and the amount recovered upon cell solubilisation at the end of each experiment).

### 4.7. Western Blotting to Quantify ERK1/2 Phosphorylation and SNAP-25 Cleavage 

Cells were washed with HBS, dissolved into LDS sample-buffer and incubated at 95 °C for 5 min. prior to electrophoresis on 12% BOLT™ polyacrylamide SDS gels. Proteins were transferred to PVDF membrane, using a Pierce Power Blotter (Thermo Fisher) according to the prescribed protocol. Non-specific binding to membranes was inhibited by incubation for 30 min. with 3% BSA in 50 mM Tris, 150 mM NaCl, 0.1% Tween-20^®^ pH 7.6. The membrane was then probed with three different antibodies, the first being selective for phosphorylated ERK (raised in rabbit, 1:1000), the second recognising both phosphorylated and non-phosphorylated (i.e., total) ERK (raised in rabbit, 1:1000), and a third that binds to SNAP-25 (mouse monoclonal, 1:3000) for either 1 h. at room temperature or overnight at 4 °C. Following additional wash steps, the membranes were exposed to AP-conjugated secondary antibody (1:10,000) for 1 h. at room temperature. Bound immunoglobulins were visualised by the development of a coloured product during incubation with 5-bromo-4-chloro-3-indolyl phosphate (0.17 mg/mL), and nitro-blue tetrazolium chloride (0.33 mg/mL) in substrate buffer (100 mM Tris, 100 mM NaCl, 5 mM MgCl_2_, pH 8.5). Images of the membrane were digitised using G: BOX Chemi-16 digital camera; their densitometric analysis was performed using ImageJ software, with the resultant data normalised to internal controls (ERK1/2 or total SNAP-25).

### 4.8. Quantification of Total Protein Amounts

Quantification of the amounts of total protein was performed by bicinchoninic acid protein assay kit against standards of BSA. TGNs were lysed in HBS supplemented with 1% (*v*/*v*) Triton X-100 on ice and 25 μL of each sample or BSA standard were applied to a 96 microplate; 200 μL mixture of reagent A and B (50:1) supplied in the kit were added to each well. The plate was mixed thoroughly and incubated for 30 min. at 37 °C before absorbance at 562 nm was read on spectrophotometer. Concentrations were calculated from the linear range of the standard curve generated in GraphPad Prism 9.

### 4.9. Quantification of TRPV1-Expressing Neurons after NGF Starvation

TGNs were grown on coverslips as in 4.4. After maintenance for 2 DIV in the presence of 50 ng/mL NGF, they were starved of neurotrophin (as in 4.3) for a further 2 DIV before fixation with 3.7% formaldehyde. Cultures were then stained with a wheat germ agglutinin-Alexa Fluor 633 conjugate (Thermo Fisher Scientific, W21404, 1:200) to aid identification of neurons before permeabilization with 0.5% Triton X-100 and immuno-labelling with anti-TRPV1 antibodies raised in guinea pig (Millipore, AB5566, 1:500). Bound primary antibodies were detected using goat anti-species IgG secondary antibodies conjugated to Alexa Fluor 488. Stained coverslips were mounted with ProLong™ Glass Antifade Mountant (Thermo Fisher Scientific) and imaged on a confocal microscope (Zeiss Observer Z1-LSM710). Images were acquired through 40× oil objective (EC Plan-NEOFLUAR 40×/1.3 NA) using Zen Black 2.3 software (Carl Zeiss, Oberkochen, Germany). The proportion of neurons expressing TRPV1 was counted manually.

### 4.10. Data Analysis

Data were calculated in MS Excel and graphs generated in GraphPad Prism 9; each point or bar represents a mean value and all error bars signify standard error of the mean (s.e.m.) as indicated in Figure legends. Welch unpaired *t*-test or one-way analysis of variance (ANOVA) with post hoc tests for comparisons between individual points were used to evaluate the significance of changes. Statistical significance was attributed to differences between groups when *p* < 0.05. Asterisks indicate *p* values; ****, *p* < 0.0001; ***, *p* < 0.001; **, *p* < 0.01; *, *p* < 0.05.
